# Prebiotic Supplementation during Lactation Affects Microbial Colonization in Postnatal-Growth-Restricted Mice

**DOI:** 10.3390/nu15122771

**Published:** 2023-06-16

**Authors:** Lucie Marousez, Léa Chantal Tran, Edwina Micours, Matthieu Antoine, Frédéric Gottrand, Jean Lesage, Delphine Ley

**Affiliations:** 1 Univ. Lille, Inserm, CHU Lille, U1286—INFINITE—Institute for Translational Research in Inflammation, F-59000 Lille, France; lea.tran@chu-lille.fr (L.C.T.); edwina.micours-@inserm.fr (E.M.); frederic.gottrand@chru-lille.fr (F.G.); jean.lesage@univ-lille.fr (J.L.); delphine.ley@chu-lille.fr (D.L.); 2 CHU Lille, Division of Gastroenterology, Hepatology and Nutrition, Department of Paediatrics, Jeanne de Flandre Children’s Hospital, F-59000 Lille, France; matthieu.antoine@chu-lille.fr

**Keywords:** prebiotic fiber, PNGR, maturation, microbial colonization, short-chain fatty acids

## Abstract

Background: An inadequate perinatal nutritional environment can alter the maturation of the intestinal barrier and promote long-term pathologies such as metabolic syndrome or chronic intestinal diseases. The intestinal microbiota seems to play a determining role in the development of the intestinal barrier. In the present study, we investigated the impact of consuming an early postnatal prebiotic fiber (PF) on growth, intestinal morphology and the microbiota at weaning in postnatal-growth-restricted mice (PNGR). Methods: Large litters (15 pups/mother) were generated from FVB/NRj mice to induce PNGR at postnatal day 4 (PN4) and compared to control litters (CTRL, 8 pups/mother). PF (a resistant dextrin) or water was orally administered once daily to the pups from PN8 to PN20 (3.5 g/kg/day). Intestinal morphology was evaluated at weaning (PN21) using the ileum and colon. Microbial colonization and short-chain fatty acid (SCFA) production were investigated using fecal and cecal contents. Results: At weaning, the PNGR mice showed decreased body weight and ileal crypt depth compared to the CTRL. The PNGR microbiota was associated with decreased proportions of the Lachnospiraceae and Oscillospiraceae families and the presence of the Akkermansia family and Enterococcus genus compared to the CTRL pups. The propionate concentrations were also increased with PNGR. While PF supplementation did not impact intestinal morphology in the PNGR pups, the proportions of the Bacteroides and Parabacteroides genera were enriched, but the proportion of the Proteobacteria phylum was reduced. In the CTRL pups, the Akkermansia genus (Verrucomicrobiota phylum) was present in the PF-supplemented CTRL pups compared to the water-supplemented ones. Conclusions: PNGR alters intestinal crypt maturation in the ileum at weaning and gut microbiota colonization. Our data support the notion that PF supplementation might improve gut microbiota establishment during the early postnatal period.

## 1. Introduction

The concept of the Developmental Origins of Health and Diseases (DOHaD) posits that the environmental conditions during the first 1000 days of life are critical for programming long-term health and disease [1,2]. In particular, clinical and preclinical studies using rodent models have shown that a deleterious nutritional environment during the perinatal period can notably program the occurrence of long-term metabolic diseases, such as obesity [3,4]. Recent studies emphasized that long-term intestinal health may also be programmed by perinatal period environmental conditions [5,6]. Indeed, the use of antibiotics during the early postnatal period is closely associated with the occurrence of inflammatory bowel diseases later in life through the modulation of the maturation and functioning of intestinal epithelial cells [7,8,9]. Indeed, the early postnatal period is a period of significant maturation of the digestive tract, with critical structural, functional and immune modifications associated with great evolution of the intestinal microbiota, passing from an immature neonatal intestine to an “adult” intestine [10,11,12]. We previously showed that postnatal growth restriction (PNGR) induced in mice by increasing the number of pups per litter was associated with delayed intestinal maturation and microbiota acquisition at weaning. This was associated with the establishment of a pro-inflammatory state that increases susceptibility to DSS-induced chronic colitis in adulthood [13]. Yet, it has been shown that the acquisition of an appropriate microbiota during weaning is essential for the maturation of the intestinal immune system, which is of major importance for the acquisition of a competent intestinal immunity and long-term intestinal health [14,15].

Thus, prevention strategies aimed at reducing the later risk of intestinal diseases due to an early deleterious nutritional environment must be developed. Prebiotics are promising candidates, given their beneficial effects on the intestinal barrier and the immune system, implemented by positively influencing the intestinal microbiota composition. Prebiotics are substrate-selectively utilized by host microorganisms to confer health benefits [16]. As they are not (fully) digested and/or absorbed in the small intestine, they reach the colon, where they can stimulate the growth of certain bacteria [17]. They display beneficial health effects through the modulation of the microbiota composition, specifically by increasing the proportions of favorable bacteria which enable the secretion of the microbial metabolites’ short-chain fatty acids (SCFAs), such as butyrate, known to favor intestinal barrier function [17,18]. Moreover, studies on animals and on healthy infants have shown that prebiotics, such as fructo- and galacto-oligosaccharides (FOS/GOS), have a long-term impact on the composition of the gut microbiota through the stimulation of the growth of beneficial bacteria and the increase in SCFA production [19,20]. Considering this knowledge, we hypothesized that early-life supplementation with prebiotics might improve gut maturation, microbial colonization and health in the context of neonatal intestinal immaturity. Thus, we investigated whether the early postnatal consumption of a resistant dextrin could impact growth, intestinal morphology and the microbiota at weaning in the context of PNGR in rodents.

## 2. Materials and Methods

### 2.1. Animals and Experimental Design

**Figure 1:**
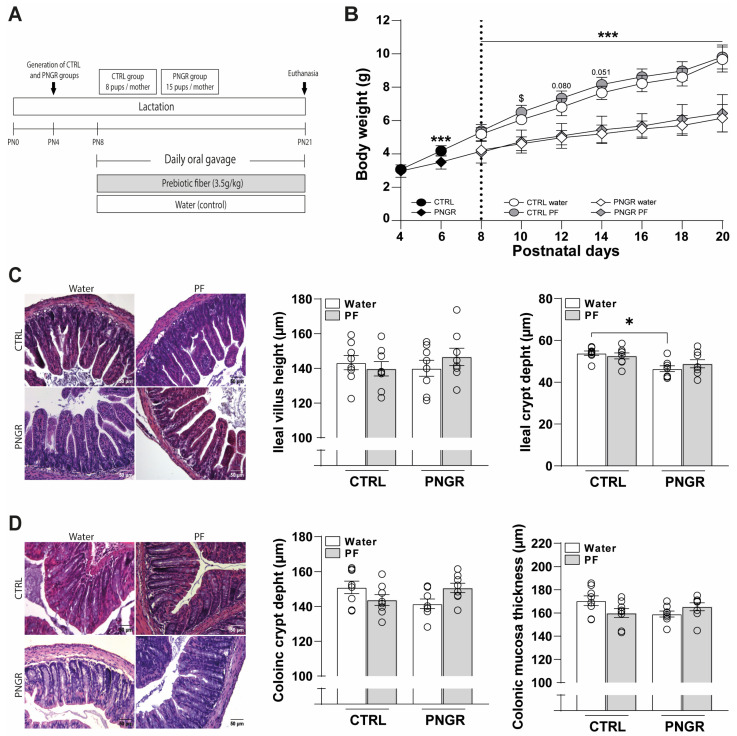
PNGR decreased pups’ growth and ileal crypt depth. (**A**) Control (CTRL, *n* = 8 pups/dam) and postal-growth-restricted (PNGR, *n* = 15 pups/dam) litters were induced at postnatal day 4 (PN4). From PN8 to PN20, the pups were supplemented with prebiotic fiber (PF) or water once daily and were euthanized at PN21. (**B**) Body weight of the CTRL water, CTRL PF, PNGR water and PNGR PF male pups during suckling (*n* = 9–20 from 2–3 litters, mean ± SD, *** *p* < 0.001 PNGR vs. CTRL; $ *p* < 0.05 PF vs. water according to mixed-effects analysis). (**C**) Histological analysis (representative images, villus height and crypt depth) of ilea from the CTRL water, CTRL PF, PNGR water and PNGR PF male pups at PN21 (*n* = 8/group, mean ± SEM, * *p* < 0.05 PNGR water vs. CTRL water by Dunn’s post Kruskal–Wallis test). (**D**) Histological analysis (representative images, crypt depth and mucosa thickness) of colons from CTRL water, CTRL PF, PNGR water and PNGR PF male pups at PN21 (*n* = 8/group, mean ± SEM).

FVB/NRj mice aged 8 weeks (Janvier Labs, Le Genest-Saint-Isle, France) were housed in a specific pathogen-free environment (12:12-h light-dark cycle) and had ad libitum access to food and water. After 7 days of acclimation, 1 male mouse was mated with 2 to 3 females for 7 days. The pregnant mice were fed with a breeding diet (SAFE^®^ diets R03-25, Augy, France). The PNGR model was induced by increasing the litter size (8 or 15 pups per litter), as described by Ley et al. [13]. Briefly, at postnatal day 0 (PN0), the litters were culled to 8 pups per mother, and those with less than 6 or more than 11 pups were excluded to homogenize lactation. At PN4, large litters of 15 pups per mother were generated by grouping pups from 2 litters of 8 pups (PNGR group, *n* = 4 litters) and further compared with control litters of 8 pups per mother (CTRL group, *n* = 5 litters) (Figure 1A). From PN8, pups from the CTRL and PNGR groups were orogastrically fed once daily with either a PF (commercial soluble-resistant dextrin, 3.5 g/kg/day) or water up to PN20. PF powder was resuspended in water (70% wt/vol) and administered as an oral solution using a lubricated polyethylene tube (0.3 mm diameter) mounted on a 30G needle. Supplementation was performed once daily in the morning from PN8 to PN20, and the feeding volume was 5 mL/kg body weight. The type of supplementation (PF or water) was randomly assigned among the litters (*n* = 2–3 litters per supplementation per group). Each pup within a litter was orally fed with the same supplementation, independent of its sex, to ensure a similar intervention within litters. The mice pups’ body weight was collected every second day until weaning (PN21) to adapt the feeding volume and validate PNGR. PNGR was validated for each pup of the PNGR group if its body weight was lower than the 10th percentile for the body weight distribution in the CTRL group from PN6 to PN21. Moreover, to avoid the sex-specific outcomes on gut barrier function observed following early life adverse environmental factors in suckling and weanling rodents [21,22], only males were studied further. At PN21, the male pups (*n* = 9–20 per group) were euthanized for sample collection. Cecal contents and colonic feces were collected with cleaned tools, immediately flash-frozen and stored at −80 °C until analysis. Ileum and colon samples were fixed in 4% paraformaldehyde overnight, processed and embedded in paraffin wax using an automatic sample preparation system (LOGOS One, Milestone Medical, Sorisole, Italy) for histological analysis.

### 2.2. Intestinal Morphology

Hematoxylin and eosin (HE) staining was performed on four-micron paraffin-embedded tissue sections. A total of 8 male pups per group were selected for histological analyses (*n* = 2–4 males per litter per group). The selections were based on the males’ body weight at PN20 to represent the average body weight and standard deviation of their litter. At PN21, the ileal villus height and crypt depth, as well as the colonic mucosa thickness and colonic crypt depth, were determined using ImageJ software v1.53t (NIH, USA) in a blinded manner by two independent observers (10 well-oriented villi or crypts per section, 9 sections from 3 segments per tissue, 8 mice per group).

### 2.3. Intestinal Microbiota

Genomic DNA (gDNA) was extracted from the colonic contents (mean weight: 14.2 g) of male pups at PN21 using an optimized tissue-specific technique, as previously described [23]. The quality and quantity of extracted gDNA were monitored via gel electrophoresis and a NanoDrop 2000 UV spectrophotometer (ThermoFisher Scientific, Illkrich-Graffenstaden, France). All gDNA samples were stored at −20 °C until further processing. The V3–V4 region of the 16S rRNA gene was amplified via PCR using the forward primer 1F (CTTTCCCTACACGACGCTCTTCCGATCT–TCCTACGGGAGGCAGCAGT) and the reverse primer 2R (GGAGTTCAGACGTGTGCTCTTCCGATCT–GGACTACCAGGGTATCTAATCCTGTT), as follows: 94 °C for 10 min, followed by 35 cycles at 94 °C for 1 min, 68 °C for 1 min and 72 °C for 1 min, with a final elongation step at 72 °C for 10 min. Amplicons were then purified using the CleanNGS magnetic beads for DNA clean-up (CleanNA). A second PCR reaction for sample multiplexing was performed using tailor-made 6 bp unique index sequences with the forward primer 2F targeting 1F (AATGATACGGCGACCACCGAGATCTACACT–CTTTCCCTACACGAC) and reverse primer 2R targeting primer 1R (CAAGCAGAAGACGGCATACGAGAT–index–GTGACT–GGAGTTCAGACGTGT), as follows: 94 °C for 10 min, 12 cycles at 94 °C for 1 min, 65 °C for 1 min and 72 °C for 1 min, with a final extension at 72 °C for 10 min. Amplicons were purified as described for the first PCR round. All libraries were pooled in the same quantity to generate an equivalent number of raw reads and were sequenced on the MiSeq Illumina platform (2 × 300 bp paired-end MiSeq kit v3, Illumina, Évry-Courcouronnes, France). The targeted metagenomic sequences were analyzed using a bioinformatics pipeline based on the ‘find, rapidly, operational taxonomic units (OTUs) with Galaxy solution’ (FROGS) guidelines [24]. The taxonomic assignment was performed using BLAST (v2.2.30) against the SILVA 138 Parc database to determine the bacterial profiles from phylum to genus and, when reachable, to species level. Alpha (Shannon and Simpson indexes) and beta diversity (Unifrac) analyses were conducted using the OTU table.

### 2.4. Dosage of Cecal Short-Chain Fatty Acids (SCFAs)

The cecal content was weighted (mean weight: 74.9 g) and homogenized in 1.5 mL of a solution of NaOH at 0.005 M, including internal standards (Acetate-D3, Propionate-D2, Butyrate-13C2 and Valerate-D9) using Precellys equipment. Total DNA was extracted following the described steps: 300 μL of supernatant was collected and transferred to a 5 mL glass tube; next, 500 μL of propanol/pyridine mix (3:2 *v*/*v*) was added and then vortexed. SCFAs were derivatized for chromatography–mass spectrometry (GC/MS) analysis using PCF. The SCFAs were extracted using 0.5 mL of hexane. The GC/MS analysis comprised a phase of liquid injection at 260 °C in the split mode and a separation phase on a 50 m × 0.25 mm, 0.25 μm, DB-5 ms capillary column. Quantification was performed with a single quadripole using electron impact ionization.

### 2.5. Statistical Analysis

Statistical analyses, except for the microbiota, were performed with GraphPad Prism 8.0 Software (San Diego, CA, USA). Variables were expressed using the mean and standard error of the mean (SEM) or standard deviation (SD). Statistical analyses were conducted based on values obtained for each individual. Outliers were excluded using Grubb’s test. The variables’ normality was assessed using the D’Agostino–Pearson test. If necessary, a log10 transformation was applied to reach normality. Thus, depending on the variable normality test results, statistical differences were tested via mixed-effects analysis (Tukey’s post-test), one-way ANOVA (Tukey’s post-test) or the Kruskal–Wallis test (Dunn’s post-test) for group comparisons. For the microbiota analysis, the normal distribution of the values was verified with the Shapiro–Wilk test. Significant variations in alpha diversity were assessed using Kruskal–Wallis or Wilcoxon rank sum tests. Multidimensional scaling analyses (MDS) were performed on beta diversity distance matrices, and differences between groups were assessed using PERMANOVA and PERMDISP analyses (2000 permutations). LEfSe (Linear discriminant analysis of Effect Size) analyses were used to determine significant variations in taxa-relative abundance [25]. Each bacterial taxon with presence in less than half of a group samples was analyzed using a chi-square test (for presence/absence analysis). Correlations were analyzed using Spearman’s r. A *p*-value < 0.05 was considered significant.

## 3. Results

### 3.1. Effects of PNGR and PF Supplementation on Pups’ Growth and Intestinal Morphology

PNGR (*n* = 15 pups) and CTRL litters (*n* = 8 pups) were created at PN4 and supplemented with water or PF daily from PN8 to weaning (Figure 1A). From PN6 to PN20, male pups from the PNGR litters displayed a significantly reduced body weight compared to CTRL pups (Figure 1B). The CTRL pups supplemented with PF tended to show a transient increased body weight from PN10 to PN14 but then displayed similar growth until PN20 compared to the CTRL pups supplemented with water. These growth patterns were representative of the evolution of body weight in each group with the sexes combined (Supplementary Figure S1).

On the ileal structure level, while villus height was not different, ileal crypt depth was reduced in the PNGR pups supplemented with water compared to the CTRL ones (Figure 1C). However, ileal crypt depth was not different between the PNGR and CTRL pups supplemented with PF, nor were colonic crypt depth or mucosa thickness significantly impacted by the PNGR model or PF administration (Figure 1D).

### 3.2. Effects of PNGR and PF Supplementation on Intestinal Microbial Colonization and SCFA Production

**Figure 2:**
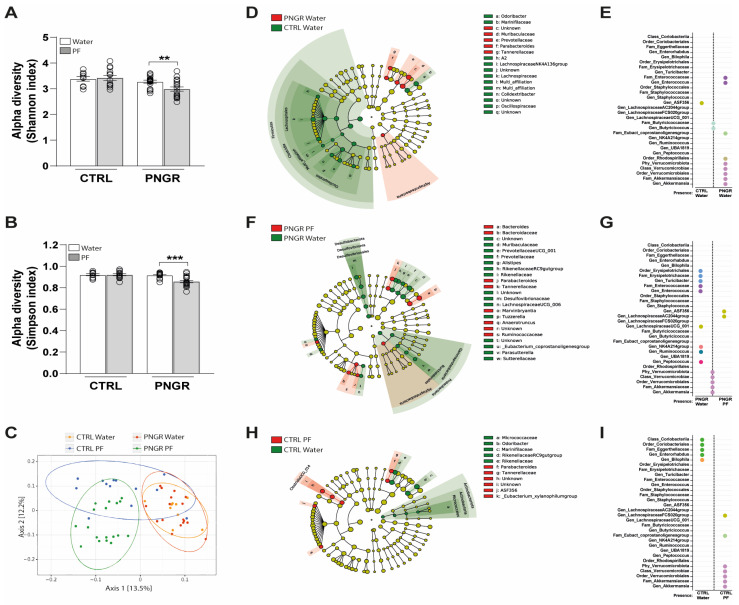
PNGR impacts gut microbial colonization, which is improved via PF supplementation. (**A**) Shannon and (**B**) Simpson indexes showing α-diversity on the OTU level in fecal samples (*n* = 9–20/group, mean ± SEM, ** *p* < 0.01 and *** *p* < 0.001 PNGR water vs PNGR PF by Dunn’s post Kruskal-Wallis test). (**C**) β-Diversity using Multidimensional scaling (MDS) ordination based on UniFrac analysis. (**D**) Difference in bacteria abundance between CTRL water and PNGR water pups represented via taxonomic cladogram following LEfSe analysis. (**E**) Presence, absence or similar proportions of specific taxonomic ranks between CTRL water and PNGR water pups following chi-square test. Dots similar colors indicate belonging to a common taxonomic rank. (**F**) Differences in bacteria abundance between PNGR water and PNGR PF pups represented via taxonomic cladogram following LEfSe analysis. (**G**) Presence, absence or similar proportions of specific taxonomic ranks between PNGR water and PNGR PF pups following chi-square test. Dots similar colors indicate belonging to a common taxonomic rank. (**H**) Difference in bacteria abundance between CTRL water and CTRL PF pups represented via taxonomic cladogram following LEfSe analysis. (**I**) Presence, absence or similar proportions of specific taxonomic ranks between CTRL water and CTRL PF pups following chi-square test. Dots similar colors indicate belonging to a common taxonomic rank.

α-Diversity was not different between the PNGR and CTRL pups, but significantly decreased microbiota richness was observed in PNGR pups supplemented with PF as compared to water (Figure 2A,B). β-Diversity, measured using the UniFrac distance, highlighted a significant difference between the four groups, mainly between the pups supplemented with PF and those supplemented with water, independent of their belonging to the CTRL or PNGR group (Figure 2C). On the phylum level, the microbiota of the CTRL and PNGR mice was mostly colonized by Bacteroidetes and Firmicutes (Supplementary Figure S2). In the CTRL pups, the microbiota was characterized by enrichment with the Lachnospiraceae and Oscillospiraceae families from the Firmicutes phylum (genera A2, Lachnospiraceae NK4A136 group and Colidextribacter) and enrichment with the Odoribacter genera (Figure 2D). Conversely, the PNGR pups’ microbiota showed an over-representation of members of the Tannerellaceae (Parabacteroides genera), Prevotellaceae and Muribaculaceae families from the Bacteroidota phylum. Interestingly, the PNGR pups were also characterized by the presence of the Akkermansia genus (Verrucomicrobiota phylum) and Enterococcus genus, which were absent in the CTRL pups (Figure 2E). In the PNGR pups, PF supplementation enriched the proportions of Bacteroides, Parabacteroides, Anaerotruncus and Marvinbryantia genera and reduced the proportions of Proteobacteria and Desulfobacterota phyla, as well as the Rikenellaceae RC9 gut group, Alistipes, Prevotellaceae UCG_001 genera and the Muribulaceae family (Figure 2F). Moreover, bacteria from the Enterococcaeae and Erysipelotrichaceae families were absent in the PNGR pups supplemented with PF as compared to water (Figure 2G). In the CTRL pups, PF administration reduced the proportions of bacteria from the Actinobacteriota phylum and Marinifilaceae and Rikenellaceae families but led to enrichment with members of the Tannerallaceae family and Clostridia UCG_014 order (Figure 2H). Moreover, while the Enterorhabdus genus was absent, the Akkermansia genus (Verrucomicrobiota phylum) was present in the PF-supplemented CTRL pups, as compared to the water-supplemented ones (Figure 2I). Finally, we found three significant correlations between ileal crypt depth and the Bacteroidota (r = −0.692, *p* < 0.001), Firmicutes (r = 0.672, *p* < 0.001) and Desulfobacterota (r = 0.367, *p* = 0.042) phyla (Supplementary Figure S3).

**Figure 3:**
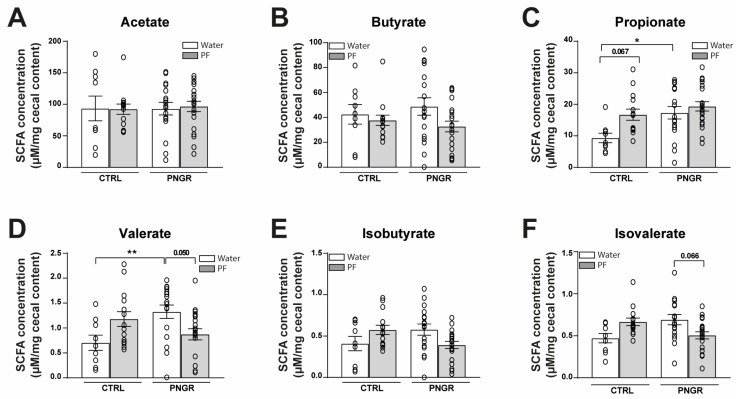
Propionate concentrations are increased with PNGR. (**A**) Acetate, (**B**) butyrate, (**C**) propionate, (**D**) valerate, (**E**) isobutyrate and (**F**) isovalerate concentrations in cecal samples between groups (*n* = 9–20/group, mean ± SEM, * *p* < 0.05, ** *p* < 0.01 according to Tukey’s test post-ANOVA or Dunn’s test post-Kruskal–Wallis).

Majors SCFAs, such as acetate and butyrate, were not affected by PNGR or PF supplementation (Figure 3A,B). However, the propionate concentrations were significantly increased in PNGR pups supplemented with water and tended to increase in CTRL pups supplemented with PF, as compared to CTRL pups supplemented with water (Figure 3C). Other less concentrated SCFAs were either increased in the PNGR pups, as for valerate (Figure 3D), or not significantly altered between groups, as for isobutyrate and isovalerate (Figure 3E,F). Finally, the propionate concentrations were positively correlated with the Akkermansia (Spearman r = 0.318, *p* = 0.016), Bacteroides (Spearman r = 0.277, *p* = 0.037) and Parabacteroides (Spearman r = 0.423, *p* = 0.001) genera (Supplementary Figure S4).

## 4. Discussion

In this study, we aimed to investigate the impact of early postnatal PF supplementation on pup growth, intestinal structure and gut microbial colonization in PNGR mice. As expected, mice in the PNGR group showed significant growth restriction during lactation, indicating severe undernutrition [13]. Surprisingly, we only found a decreased ileal crypt depth in the PNGR pups, without any impact of growth restriction on colonic structures [13]. We postulate that this discrepancy may be due to the trophic effects of glucocorticoid hormones secreted by neonatal mice in response to the chronic stress (daily oral gavage) introduced in this model. Indeed, while glucocorticoid hormones, such as corticosterone, are released in cases of chronic stress [26], hypercortisolism has been shown to promote precocious colonic maturation in young rats [27]. In addition, consistent with our previous findings, microbial colonization was substantially altered in the PNGR pups compared to the CTRL ones [13]. Indeed, the lower abundance of the Odoribacter genus and several members of Firmicutes (Lachnospiraceae and Oscillospiraceae families) and the greater abundance of the Parabacteroides genus, as well as the presence of the Enterococcus genus, in the undernourished pups are highly representative of the immature profile established by PNGR [13]. Moreover, the presence of Verrucomicrobiota in the PNGR pups is consistent with the normal evolution of this phylum during the early postnatal period [12] and the microbial colonization delay induced by the PNGR model [13]. Indeed, Akkermansia muciniphila supplementation in rodents enhanced intestinal epithelial development through the acceleration of intestinal stem cell proliferation and promoted the differentiation of Paneth cells and goblet cells in the small intestine [28]. Thus, the detection of the Akkermansia family in PNGR could be the result of its necessary presence for proper intestinal maturation during postnatal development. Moreover, the beneficial effects of Akkermansia bacteria might occur through the production of metabolic compounds, such as propionate, a health-promoting and anti-inflammatory SCFA, which was associated with Akkermansia family presence in our study and others [28,29]. In addition, Pandey et al. (2022) recently showed that intestinal mucosal integrity and maturation during mouse postnatal life is accompanied by the co-development of specific gut microbial colonization at specific time points [12]. However, in the case of early development and/or malnutrition, it is still unclear whether changes in the microbiota promote or simply result from alterations in the intestinal mucosa [30,31]. Moreover, whereas a high energy demand is required for intestinal epithelial cells during maturation [32], it is plausible that caloric and/or protein–energy restriction induced in the PNGR model, potentially associated with the lesser availability of milk bioactive factors implicated in gut maturation [33], might directly lead to growth restriction and blunted intestinal maturation. Thus, further studies are needed to elucidate the question of causality between undernutrition and histological and microbiota composition changes. Finally, our results can be related to recent studies which propose that intestinal maturation, especially through the proper establishment of the intestinal microbiota, are decisive for the maturation of the intestinal immune system, the acute inflammatory response and susceptibility to intestinal inflammatory pathologies later in life [13,14,34].

Then, we investigated whether a fermentable fiber supplementation during lactation could counter PNGR effects, particularly on microbiota colonization. We did not report significant effects of PF administration during lactation on body weight or intestinal morphology in the PNGR and CTRL groups. Similarly, other studies investigating the impacts of postnatal supplementation with different prebiotic compounds in rodents failed to induce significant body weight changes during lactation or at weaning [35,36]. Although the impact of prebiotic supplementation on intestinal microbiota colonization remains undeniable in the case of postnatal supplementation, the results can vary widely between studies [19,35,36]. Indeed, Morel et al. (2015) clearly showed that the prebiotic compound’s nature differentially impacts the composition of the microbiota in the young rat, as well as the programming the adult intestinal microbiota, making comparisons difficult [19]. Here, we propose that PF supplementation might improve gut microbial colonization in both PNGR and CTRL pups. Indeed, we showed that PF supplementation increased the proportions of beneficial bacteria from the Bacteroides and Parabacteroides genera in PNGR, which participate in gut homeostasis and inflammation regulation through the secretion of SCFAs, such as propionate [37,38]. Moreover, prebiotic supplementation in undernourished mice was associated with a lesser content of inflammatory-related and potentially pathogenic bacteria, represented by an absence of the Erysipelotrichaceae family and Enterococcus genus in this group, compared to the group supplemented with water [13,39]. Finally, the decreased proportions of Proteobacteria and particularly γ-Proteobacteria, which dominates the neonatal “immature” microbiota, in the PNGR pups supplemented with PF also support this hypothesis [40]. In the CTRL pups, the beneficial effects of PF supplementation were marked by the increased proportions of the beneficial Parabacteroides genus [37], as well as the lesser content of the Actinobacteriota phylum, the proportions of which normally decrease in the progression from postnatal age in rodents [12]. However, although we did not find any significant effects of PF supplementation on ileal and colonic histological features, the presence of the Akkermansia genus and a trend towards increased propionate concentrations were found the CTRL-PF pups compared to the CTRL-water pups. Thus, due to the positive effects of Akkermansia muciniphila and propionate on intestinal immune cell development [28,29], we hypothesized that the presence of bacteria from the Verrucomicrobiota phylum following PF supplementation in CTRL pups might be beneficial for intestinal homeostasis and maturation.

## 5. Conclusions

To conclude, this study supports the notion that early postnatal nutrition is crucial for proper gut microbial colonization and that prebiotic fiber supplementation during this critical period of development might contribute to the improvement of the protective properties in altered nutritional environments. However, the consequences of such modifications for gut microbiota during postnatal development and in response to PNGR on later intestinal health and susceptibility to chronic intestinal diseases remain to be investigated [5,41].

## Data Availability

Data from this study are available upon request from the authors.

## Supplementary Materials

Supporting information can be downloaded at: https://www.mdpi.com/article/10.3390/nu15122771/s1, Figure S1: Pups body weight in both sexes, Figure S2: Individual phylum composition, Figure S3: Phyla and crypt depth correlations, Figure S4: Propionate and genera correlations.
