# Supplementary material for: Prebiotic Supplementation during Lactation Affects Microbial Colonization in Postnatal-Growth-Restricted Mice
Source: Nutrients. 2023 Jun 16;15(12):2771. doi: 10.3390/nu15122771 (PMC10300902; doi:10.3390/nu15122771)
Supplement: Supplementary file 1 [file nutrients-15-02771-s001.zip › nutrients-2400392-supplementary.pdf]

Supplemental material

Supplemental Figure S1

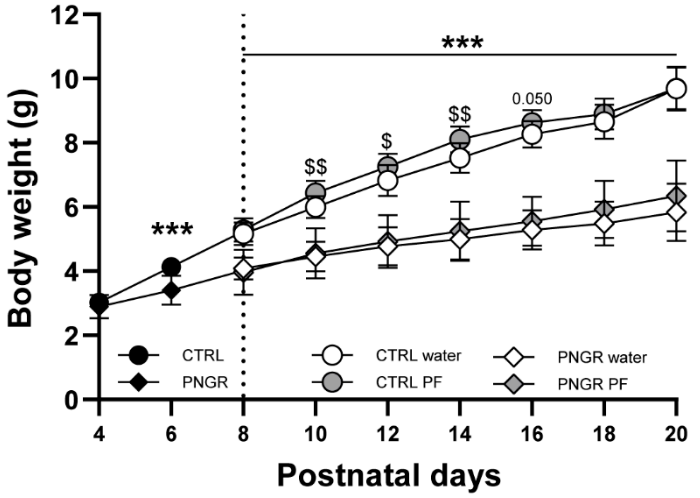

Pups body weight in both sexes. Body weight in control (CTRL) and postnatally growth restricted (PNGR) pups supplemented with water or prebiotic fiber (PF) female and male pups during suckling (n=16-30 from 2-3 litters, mean±SD, ***p<0.001 PNGR vs CTRL; $p<0.05, $$p<0.01 PF vs water by mixed-effects analysis).

Supplemental Figure S2

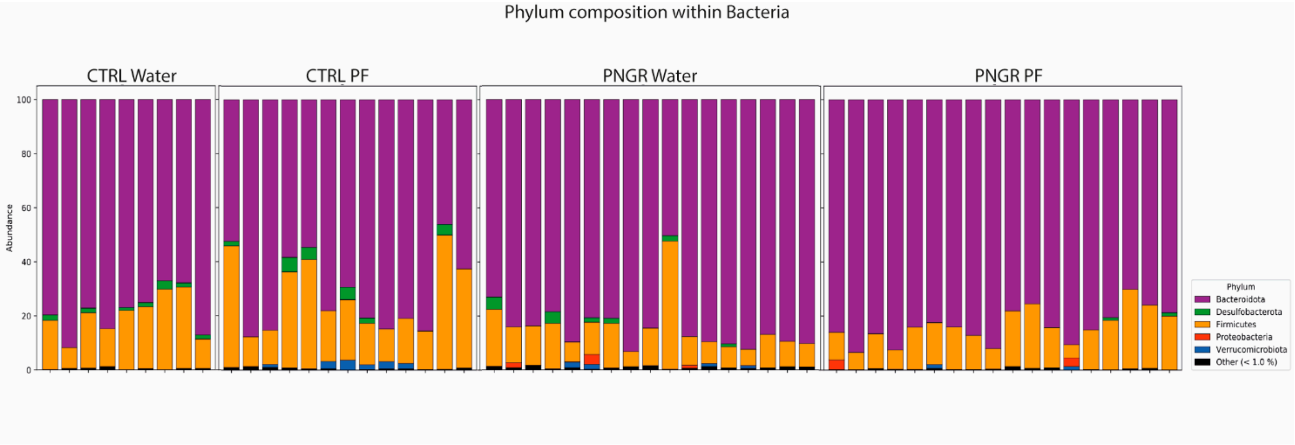

Individual phylum composition. Phylum composition within Bacteria in control (CTRL) and postnatally growth restricted (PNGR) pups supplemented with water or prebiotic fiber (PF) of male pups at postnatal day 21 (PN21). n=9-20 from 2-3 litters.

Supplemental Figure S3

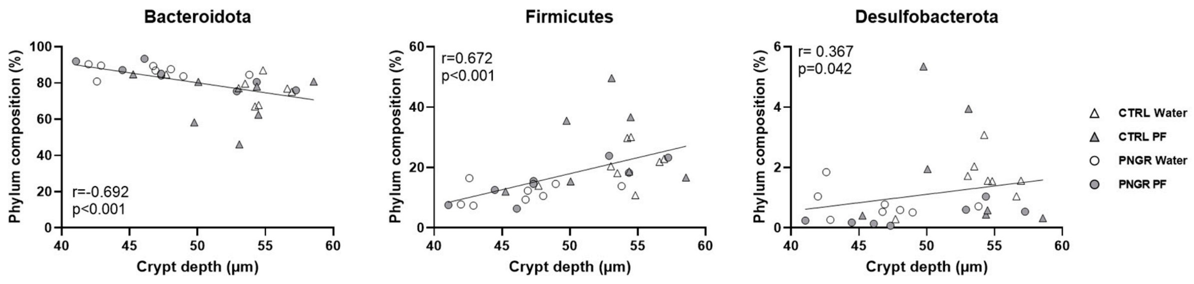

Phyla and crypt depth correlations. Spearman correlations between ileal crypt depth and Bacteroidota, Firmicutes and Desulfobacterota phyla in control (CTRL) and postnatally growth restricted (PNGR) male pups supplemented with water or prebiotic fiber (PF) at postnatal day 21 (PN21). n=9-20 from 2-3 litters.

Supplemental Figure S4

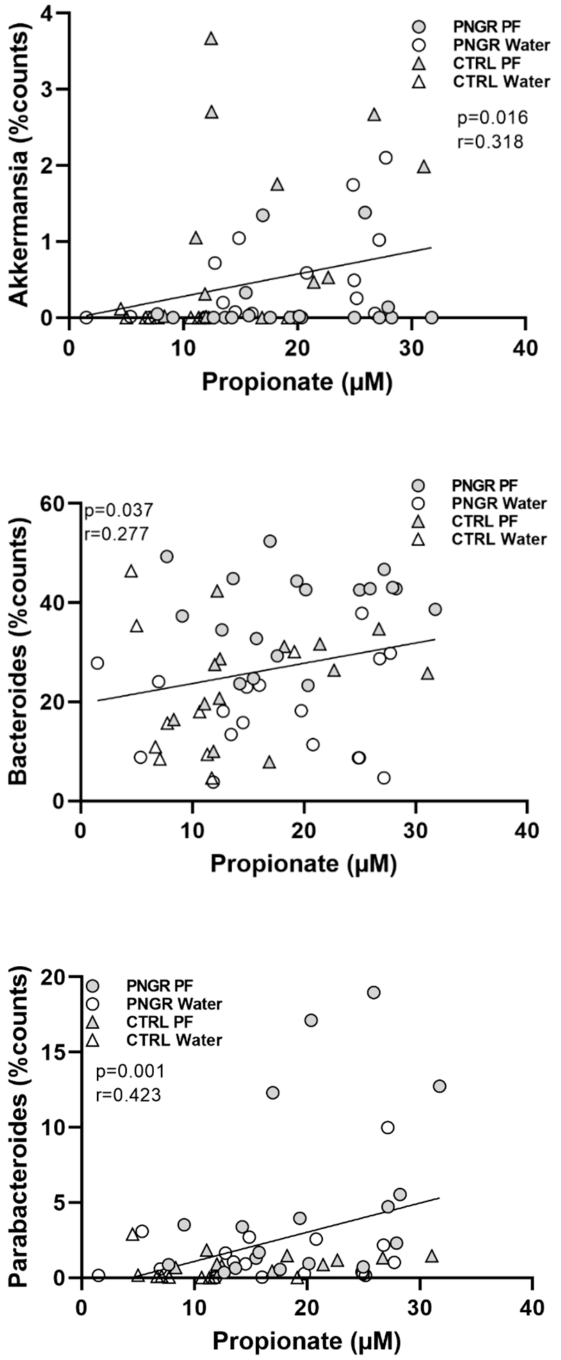

Propionate and genera correlations. Spearman correlations between cecal concentrations of propionate and Akkermansia, Bacteroides and Parabacteroides genera content of fecal samples in control (CTRL) and postnatally growth restricted (PNGR) male pups supplemented with water or prebiotic fiber (PF) at postnatal day 21 (PN21). n=9-20 from 2-3 litters.
